# The tapetal AHL family protein TEK determines nexine formation in the pollen wall

**DOI:** 10.1038/ncomms4855

**Published:** 2014-05-08

**Authors:** Yue Lou, Xiao-Feng Xu, Jun Zhu, Jing-Nan Gu, Stephen Blackmore, Zhong-Nan Yang

**Affiliations:** 1 College of Life and Environment Sciences, Development Center of Plant Germplasm Resources, Shanghai Normal University, Shanghai 200234, China; 2 Royal Botanic Garden Edinburgh, 20a, Inverleith Row, Edinburgh EH3 5LR, UK; 3 These authors contributed equally to this work

## Abstract

The pollen wall, an essential structure for pollen function, consists of two layers, an inner intine and an outer exine. The latter is further divided into sexine and nexine. Many genes involved in sexine development have been reported, in which the MYB transcription factor Male Sterile 188 (MS188) specifies sexine in *Arabidopsis*. However, nexine formation remains poorly understood. Here we report the knockout of *TRANSPOSABLE ELEMENT SILENCING VIA AT-HOOK* (*TEK)* leads to nexine absence in *Arabidopsis*. *TEK* encodes an AT-hook nuclear localized family protein highly expressed in tapetum during the tetrad stage. Absence of nexine in *tek* disrupts the deposition of intine without affecting sexine formation. We find that ABORTED MICROSPORES directly regulates the expression of *TEK* and *MS188* in tapetum for the nexine and sexine formation, respectively. Our data show that a transcriptional cascade in the tapetum specifies the development of pollen wall.

## Abstract (web summary)

The nexine is a conserved layer of the pollen wall in land plants. The authors show that the AHL family protein TRANSPOSABLE ELEMENT SILENCING VIA AT-HOOK (TEK) is necessary for nexine formation in *Arabidopsis*, acting downstream of the transcription factor ABORTED MICROSPORES (AMS).

## Graphical abstract

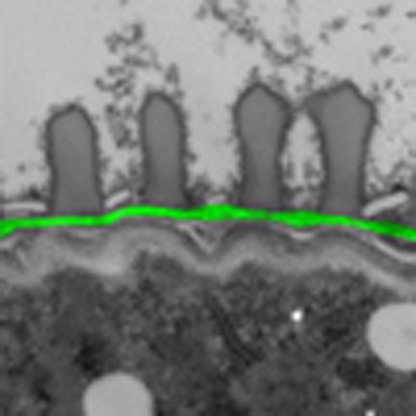

The multilayered, structurally complex walls typical of angiosperm pollen grains exhibit a greater level of organizational complexity than those of any other cell type^1^. The main roles of the pollen wall are in guiding the male gametophyte development in anthers, protecting the pollen from various environmental stresses and functioning in cell–cell recognition during pollination^1^,^2^,^3^. The vast morphological diversity exhibited by pollen walls is the basis of the discipline of palynology and there is much interest in understanding how this diversity has arisen^4^. The pollen wall consists of a specialized outer exine, composed of sporopollenin, and an inner cellulosic intine. Sporopollenin is highly resistant to physical, chemical and biological degradation^5^,^6^. Based on cytological and molecular evidence, tapetum fills the role of sporopollenin biosynthesis for exine formation^7^,^8^,^9^,^10^,^11^,^12^. In angiosperms, the pollen exine is divided into sexine and nexine. The non-sculptured nexine is distinguished as a distinct layer between the sculptured sexine and an inner intine, which appears to be strongly conserved in seed plants^13^,^14^,^15^. However, apart from its morphological description, knowledge on the formation of the nexine and its function is rather meagre. Therefore, the recognition of nexine formation in pollen wall development not only provides insight into plant phylogeny but also reveals the gene-determined mechanisms that underlie the ontogeny of the major layers of the pollen wall.

Recently, well-characterized genes in which mutations cause male sterile phenotype have enriched our understanding of key events in pollen wall development. Most of them, which are highly expressed in the tapetum, are required for exine formation. *In vitro* enzyme activity analysis revealed that they synthesize polymers such as sporopollenin precursors^16^,^17^,^18^,^19^,^20^,^21^,^22^. The regulatory factors required for tapetum development and function are also important for the pollen wall formation^11^,^12^,^23^. Among them, five transcription factors form a genetic pathway (DYT1-TDF1-AMS-MS188-MS1) that regulates tapetum development and function^24^. In this pathway, *DYSFUNCTIONAL TAPETUM1* (*DYT1*) encodes a bHLH transcription factor; *DEFECTIVE in TAPETAL DEVELOPMENT and FUNCTION1* (*TDF1*) encodes a R2R3 MYB transcription factor, which both function in the early stages of tapetum development and are essential for the expression of tapetal genes^25^,^26^. *ABORTED MICROSPORES* (*AMS*), an MYC class transcription factor, directly regulates the expression of ATP-Binding Cassette Transporter G26 (*ABCG26*, also named *WBC27*), which is a putative transporter of tetraketide sporopollenin precursors for exine formation^12^,^27^,^28^,^29^. *MS188* encodes an R2R3 MYB transcription factor that specially affects sexine formation^11^.

Here we report the identification of an *Arabidopsis* mutant *transposable element silencing via AT-hook (tek)* that is associated with the formation of the nexine layer. The *TEK* encodes an AT-hook nuclear localized (AHL) protein and is highly expressed in the tapetal layer at tetrad stage. We show that AMS in the tapetum directly regulates *TEK* and *MS188* for nexine and sexine layer formation, and that the presence of an intine layer depends on the formation of the nexine. These results establish a series of transcriptional events that eventually dictate the development of the different layers of the pollen wall.

## Results

### *TEK* knockout leads to male sterility

**Figure 1:**
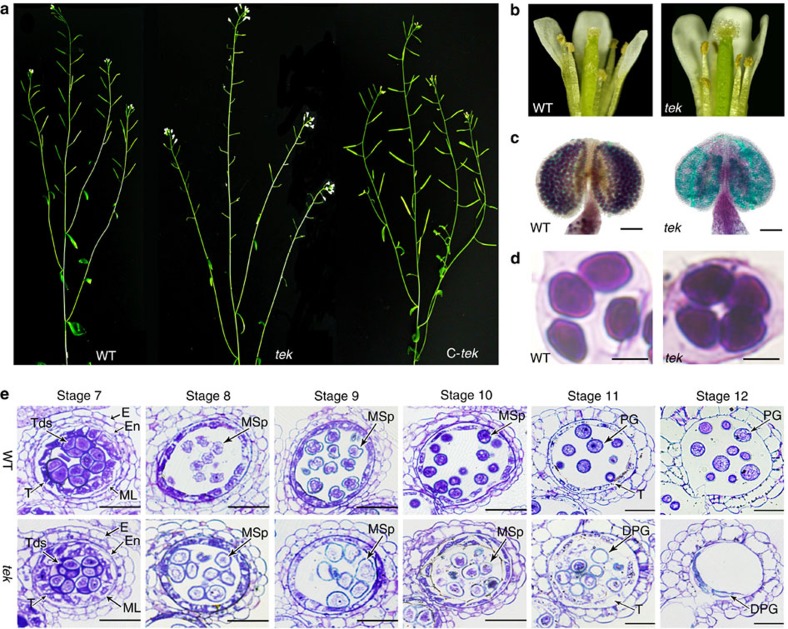
*TEK* knockout leads to the defective pollen development. (**a**) The wild type (WT), *tek* plant with short siliques (*tek*) and complemented plant with normal siliques (*C-tek*). (**b**) The flower of wild type and *tek*. (**c**) Alexander’s staining of wild-type and *tek* anther. Scale bar, 100 μm. (**d**) The tetrad of wild type and *tek*. Scale bars, 10 um. (**e**) Sections of wild type and *tek* showing anther development from stage 7–12. DPG, degenerated pollen grain; E, epidermis; En, endothecium; ML, middle layer; MSp, microspore; PG, pollen grain; T, tapetum; Tds, tetrads. Scale bars, 20 μm.

To identify genes essential for anther development, a sterile mutant was isolated in a collection of T-DNA-tagged lines^30^. The T-DNA is inserted in At2g42940 (see below), which was previously designated as *TRANSPOSABLE ELEMENT SILENCING VIA AT-HOOK* (*TEK*). Knockdown of TEK led to late flowering^31^. Here we focus on the mechanism of the male sterility of *tek*. The *tek* mutant was indistinguishable from wild type during vegetative growth, by its short siliques without seeds (Fig. 1a). No pollen was observed on the stamens and stigma of flowers (Fig. 1b) and Alexander’s staining showed that all pollen grains in the locule were aborted during the late stages of anther development (Fig. 1c). Each mutant tetrad contained four microspores that were similar to those of wild type, indicating that meiosis is normal in *tek* (Fig. 1d). Reciprocal crosses with wild type indicated that female fertility was not affected in the *tek* mutant. The fertile and sterile plants of F2 population segregated with 3:1 (281:95) ratio, indicating a single recessive sporophytic mutation for *tek*.

Anther development in *Arabidopsis* can be divided into 14 well-ordered stages on the basis of morphological landmarks^32^. At stage 8, the microspores are released from tetrads. The *tek* microspores were rounder and larger than those of wild type. At stage 9, microspores of wild-type plant became vacuolated, whereas the cytoplasm of *tek* microspores was shrunken and disintegrated. During stage 10–12, microspores of wild type underwent asymmetric mitotic divisions and gradually developed into mature pollen grains. In the mutant, the microspore cytoplasm further degenerated and finally all microspores were aborted at stage 12 (Fig. 1e). These results show that the defective microspore production during microgametogenesis causes the complete male sterility of *tek* plant.

### Nexine and intine layers are absent in *tek*

**Figure 2:**
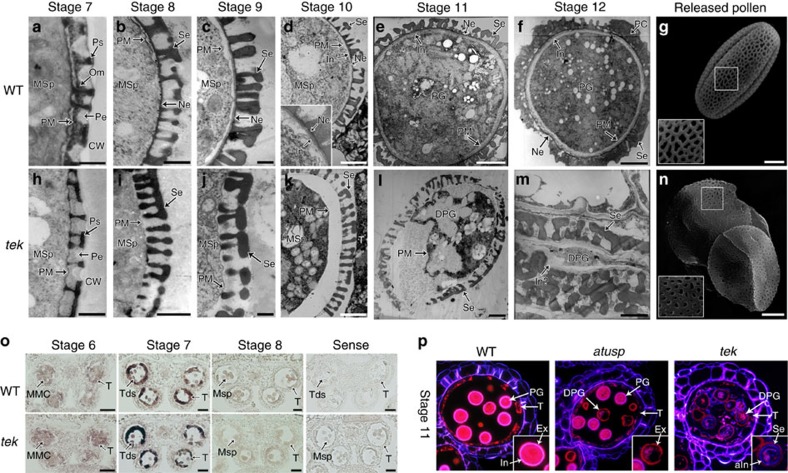
Nexine and intine layers are absent in *tek* (a–n). TEM images of pollen wall development in wild type (**a**–**f**) and *tek* (**h**–**m**) from stage 7–12. (**a**,**h**) Later stage 7, showing the osmiophilic materials is reduced in *tek*; (**b**,**i**), stage 8, showing the nexine layer is absent in *tek*; (**c**,**j**), stage 9; (**d**,**k**), stage 10, showing the intine commenced in wild type; (**e**,**l**), stage 11, showing the degenerated *tek* pollen grain; (**f**,**m**), stage 12, showing the sexine and material analogues of intine still visible in *tek*. Scale bars, 500 nm (**a**–**c** and **h**–**j**); scale bars, 2 μm (**d**–**f** and **k**–**m**). Scanning electron microscopy of pollen grain of wild-type (**g**) and *tek* (**n**) showed the similar reticulate pattern. Scale bars, 5 μm. (**o**) AtUSP had its similar expression pattern in wild-type and *tek* anthers. Scale bars, 20 μm. (**p**) Cytochemical staining of semi-thin sections of wild-type, *atusp*/+ and *tek*. All wild-type pollens showed a pink fluorescent ring of the intine layer, while the half pollens of *atusp*/+ showed the absence of intine layer. A dim blue fluorescent ring was detected in the degraded pollen of *tek*. aIn, abnormal intine; Ca, callose wall; DPG, degenerated pollen grain; In, intine; In?, intine-like materials; MMC, microspores mother cell; MSp, microspore; Ne, nexine; PC, pollen coat; Pe, primexine; PG, pollen grain; PM, plasma membrane; Ps, pro-sexine; Se, sexine; T, tapetum; Tds, tetrads.

The pollen wall pattern is determined by several essential events at tetrad stage, including callose deposition, primexine matrix secretion and plasma membrane undulation^33^,^34^,^35^,^36^,^37^. These events were indistinguishable between wild type and *tek*. The pro-sexine could be observed in both wild type and *tek*. However, accumulation of osmiophilic materials was significantly reduced in periplasmic space on the surface of microspores in *tek* at later tetrad stage (Fig. 2a,h). When callose wall is totally dissolved at stage 8, the nexine layer was clearly present in wild type (Fig. 2b) but completely lacking in the *tek* microspores (Fig. 2i). In wild-type microspores, the intine layer between the nexine and the microspore plasma membrane could be clearly detected at stage 10 (Fig. 2d), and continues to increase in thicknesses as the pollen grains mature (Fig. 2e,f). In *tek* anthers, both the nexine and intine were absent despite the formation of a normal sexine (Fig. 2j). Later, the lack of nexine and intine in *tek* was more distinct (Fig. 2k,l). By stage 12, all pollen grains of the mutant had collapsed, with the framework of the sexine still visible. Some materials with low electronic density were also observed under the sexine layer (Fig. 2m). The surface pattern of the sexine layer in *tek* was similar to that of the wild type (Fig. 2g–n), although the shape of the reticulum was slightly different, perhaps as a result of pollen collapse. These results show that the *tek* mutant failed to develop both nexine and intine, causing microspore abortion and male sterility.

### Intine deposition is dependent on the presence of nexine

Intine development is gametophytically controlled^38^,^39^,^40^. To understand how the sporophytic gene *TEK* affects the development of intine, we analysed the expression of an intine related gene in *tek*. *Arabidopsis* UDP-sugar pyrophosphorylase (AtUSP) was reported to play a critical role in intine synthesis^40^. The expression level of *AtUSP* in *tek* was similar to that of wild type (Supplementary Table 1). *In situ* hybridization confirmed that the expression pattern of *AtUSP* was not affected in *tek* (Fig. 2o). We used Tinopal and diethyloxadicarbocyanine iodide (DiOC_2_) to stain the intine and exine, respectively (Fig. 2p). In wild type, the intine showed a distinct fluorescent ring between the exine and cytoplasm of pollen. In the locule of *atusp*/+, half of the microspores (*atusp*) are abnormal (Supplementary Fig. 1). The fluorescent ring of intine was not observed in the *atusp* pollen (Fig. 2p). In *tek*, a dim fluorescent ring (aIn) appeared between exine and cytoplasm of pollen. It is likely to be that the materials required for intine formation can be synthesized in *tek* microspores. However, the deposition of intine is affected by the absence of nexine in *tek* microspores, suggesting that intine deposition relies on the presence of the nexine layer.

### *TEK* is highly expressed in tapetal cells at tetrad stage

**Figure 3:**
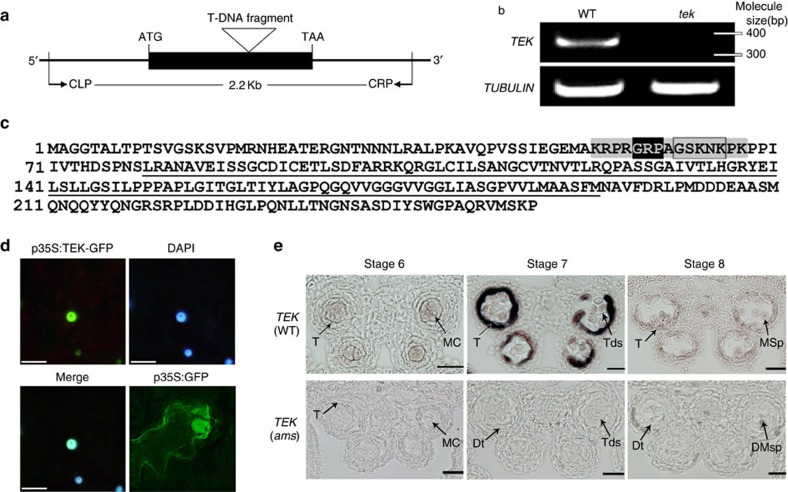
*TEK* is highly expressed in tapetal cells in anther. (**a**) *TEK* (At2g42940) contained only one exon (black box). The T-DNA insertion site was indicated and DNA fragment for complementation was outlined by primers CLP and CRP. (**b**) The *TEK* transcript could not be detected in *tek* inflorescence. (**c**) Amino acid sequence of TEK contained the PPC domain (underlined) and the AT-hook motif (grey) within GRP motif (black) and type I AT-hook motif (boxed); (**d**) the fluorescence co-localization of p35S:TEK-GFP and DAPI (4',6-diamidino-2-phenylindole) indicated TEK was located to the nuclear. The p35S:GFP was used as a control. Scale bars, 10 μm. (**e**) *TEK* transcript was mainly detected in tapetal cell at stage 7 in wild-type, whereas this signal could not be detected in *ams* mutant. Stage 5–7, scale bars, 20 μm; Stage 8–11, scale bars, 50 μm.

To ascertain the insert location of T-DNA tag, thermal asymmetric interlaced TAIL–PCR^41^ was carried out and a genomic DNA fragment flanking the left border of T-DNA was recovered. Sequencing of the TAIL–PCR product showed that the T-DNA was inserted in the only exon of At2g42940, which completely abolished the expression of At2g42940 (Fig. 3b). For genetic complementation, we cloned the 2,277-bp genomic region, including the promoter and 3′-untranslated region of At2g42940 from wild type and transformed it into the *tek/+* heterozygous plants. Of the 20 transgenic plants with *tek*/*tek* genotype, 19 plants showed the normal fertility (Fig. 1a). Our results demonstrated that At2g42940 was *TEK* and the phenotypes were caused by the disruption of At2g42940.

*TEK* encodes an AHL protein with a type I AT-hook motif for matrix attachment region binding and a PPC (Plants and Prokaryotes Conserved) domain evolutionarily conserved in the plant kingdom (Fig. 3c). In *Arabidopsis*, AHL family contains 29 members of 2 subgroups^42^ and TEK (AHL16) forms a distinct clade in subgroup I (Supplementary Fig. 2b). TEK homologues exist in both monocots and dicots. In some plants, such as rice and *Medicago*, there was only one closely related homologue, while two homologues were identified in *Populus* and soybean (Supplementary Fig. 2c). *Arabidopsis* AHL family was reported to be located in the nucleus^43^. We transformed tobacco leaves with p35S:*TEK*-GFP and found that the fluorescence of the TEK-GFP protein was co-localized with the 4′,6-diamidino-2-phenylindole staining in nuclei (Fig. 3d), indicating that TEK is localized in the nuclear region.

Reverse transcriptase–PCR (RT–PCR) analysis showed that *TEK* was widely expressed in flowers, roots and stems, with relatively low expression in leaves (Supplementary Fig. 2a). As TEK functions in anther development, we carried out *in situ* hybridization to detect its spatial and temporal expression pattern in wild-type anthers (Fig. 3e). During meiosis (stage 6), a weak hybridization signal was observed in microspore mother cells and tapetum. At the tetrad stage (stage 7), the maximum hybridization signal was seen in the tapetal cells with weak signal in the tetrads. At stage 8, with the microspores released from the tetrad, *TEK* expression was dramatically reduced in both tapetum and microspores. Thereafter, a hybridization signal was no longer observed. The expression pattern of *TEK* is in agreement with its function for pollen wall formation during anther development.

### AMS directly binds the promoters of *TEK* and *MS188*

**Figure 4:**
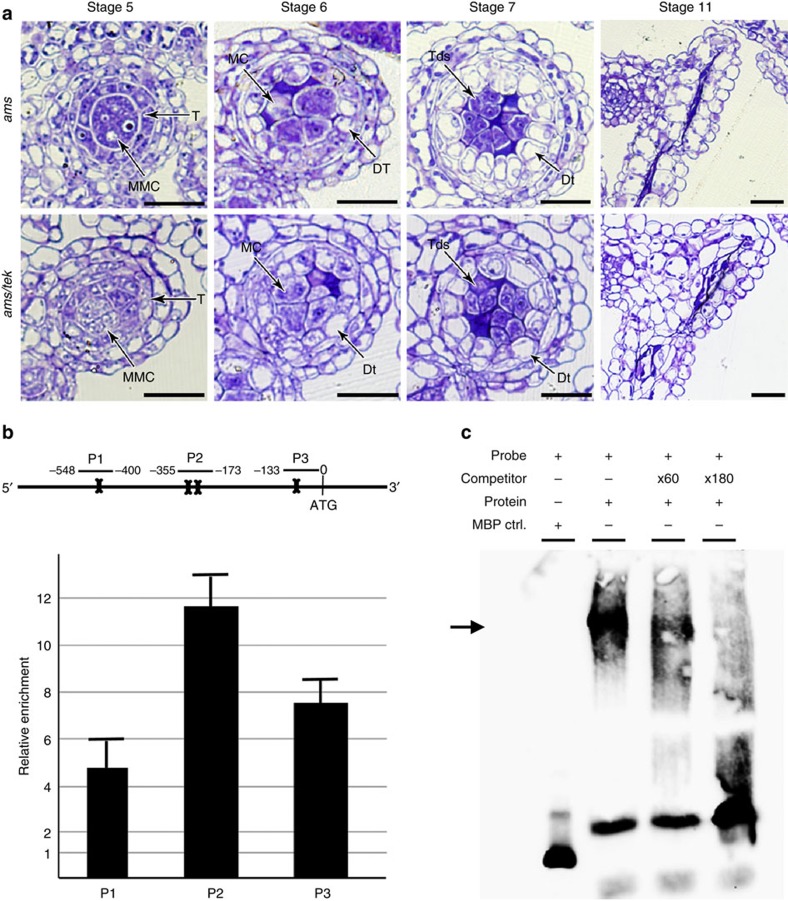
AMS directly binds to the promoter of *TEK*. (**a**) Sections of anther development show the phenotype of *ams tek* double mutant is similar with *ams*, indicating *TEK* functions downstream of *AMS*. DMSp, degenerated microspores; Dt, degenerated tapetum; MC, meiotic cell. Scale bars, 20 μm. (**b**) The *TEK* promoter region contains four predicted E-boxes (bone). The enrichments of *TEK* promoter were confirmed by ChIP–quantitative PCR (qPCR) with the primer sets (P1, P2, P3). Fold enrichment calculations from two replicates qPCR assays in three independent ChIP experiments. Error bars represent s.d. (*n*=3). (**c**) EMSA assay shows AMS can bind to P2 fragment in the *TEK* promoter. Non-labelled competitors are able to reduce the visible shift significantly (arrow).

In the tapetum of *Arabidopsis*, there is a genetic pathway of DYT1-TDF1-AMS- MYB103-MS1, which is essential for tapetum development and function^24^. As *TEK* is mainly expressed in the tapetum at stage 7 (Fig. 3e), we performed *in situ* hybridization using *TEK* probe to further understand its position of the genetic pathway. We found that *TEK* signal could not be detected in *ams* anther, whereas the expression of *TEK* is not affected in *ms188*, supporting the hypothesis that *TEK* expression was under the regulation of AMS (Fig. 3e, Supplementary Fig. 4). We constructed double mutant of *ams tek* (Supplementary Fig. 3a,b). The *ams tek* plant showed male sterile phenotype with normal vegetative growth (Supplementary Fig. 3c). The defective phenotype of tapetal development in the double mutant was similar to *ams* (Fig. 4a). These results show that *TEK* genetically acts downstream of *AMS* in the tapetum.

To analyse whether *AMS* directly regulates *TEK* expression *in vivo*, we performed chromatin immunoprecipitation (ChIP) analysis. AMS was reported to bind to the sequence containing E-box (CANNTG)^12^. There are four E-box motifs in the *TEK* promoter region (−29 to −24, −236 to −231, −262 to −257 and −506 to −501) (Fig. 4b). Polyclonal antibodes against AMS was used for ChIP with wild-type inflorescence. Enrichments were detected with three primer sets in *TEK* promoters, showing that *TEK* was positively enriched in +AB (antibody) compared with –Ab samples (Fig. 4b). Electrophoretic mobility shift assay (EMSA) was further performed to confirm the quantitative ChIP–PCR results *in vitro*. The full-length AMS was cloned into the pMAL plasmid and the recombinant AMS protein was purified from the supernatant. AMS protein can bind to the *TEK* promoter fragment (−294 to −12) with the supershift band. A 60-fold and a 180-fold excess of unlabelled *TEK* promoter fragment gradually competed for AMS and significantly reduced the supershifted band. In the negative control, no parallel shift band was detected with the maltose binding protein (MBP) tag only (Fig. 4c). These data show that AMS directly binds to the E-box regions in *TEK* promoter, suggesting AMS might regulate *TEK* expression for nexine formation.

**Figure 5:**
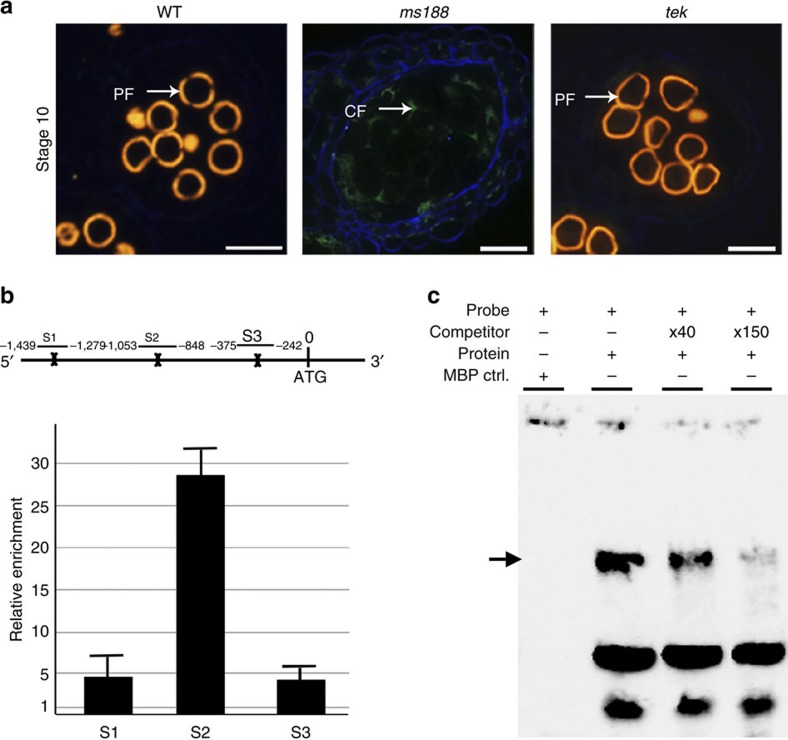
AMS directly binds to the promoter of *MS188*. (**a**) Cytochemical staining of anther sections show autofluorescence of sexine in wild-type and *tek* but not in *ms188*. CF, callose fluorescence; PF, pollen wall autofluorescence. Scale bars, 20 μm. (**b**) The enrichments of *MS188* promoter were confirmed by ChIP–quantitative PCR (qPCR) with the primer sets (S1, S2, S3), suggesting *MS188* is a direct target of AMS. Fold enrichment calculations from two replicates qPCR assays in three independent ChIP experiments. Error bars represent s.d. (*n*=3). (**c**) EMSA assay shows AMS is able to bind to S2 fragment in the *MS188* promoter. Non-labelled competitors are able to reduce the visible shift significantly (arrow).

Sexine autofluorescence can be directly observed under the ultraviolet light. In wild-type and *tek*, the autofluorescence was clearly observed, indicating the existence of the sexine layer. However, this autofluorescence can not be detected in the *ms188* mutant (Fig. 5a), further supporting the previous transmission electron microscopic (TEM) observation^11^ that the sexine layer is completely abolished in *ms188*. Previous data showed that *MS188* was not expressed in *ams* mutants, and that the phenotypes of *ms188 ams* double mutants were similar to those of *ams* instead of *ms188* (ref. 24). Using ChIP and EMSA analysis, we found that AMS can also bind to the *MS188* promoter (Fig. 5b,c). In addition, the expression of *TEK* and *MS188* was not affected by each other (Supplementary Fig. 4). Microarray data show that genes responsible for the sexine are not affected by *TEK* (Supplementary Table 1). Thus, these results suggest that sexine and nexine formation are independent, although both are under control of AMS in the tapetum.

## Discussion

Nexine is a conserved structure of the pollen wall in vascular land plants based on the observation under TEM^4^,^13^,^14^,^15^. In this work, TEM analysis showed that osmiophilic materials were accumulated in periplasmic space on the surface of microspores in wild type. Later, the nexine layer was quickly formed surrounding the microspore surface after microspore released from the tetrad. However, the nexine formation had not been detected in *tek* and sexine failed to adhere or anchor to the microspore surface. In addition, the intine was absent in *tek*; however, the intine gene expression was not affected and the materials for intine formation seemed to be synthesized. These results indicate that *TEK* acts as a regulator controlling nexine formation during pollen development. The intine formation is gametophytically controlled^38^,^39^,^40^. However, the *TEK* gene was expressed in the tapetum under sporophytic control. Thus, we suppose that the intine formation depends on the presence of the nexine, which is consistent with the fact that the intine is formed after the nexine during the pollen wall development. This work provides a potential material for further investigation of nexine formation and function.

*TEK* encodes a member of the AHL family protein. It is reported to function in the maintenance of genome integrity by silencing transposable element and repeat-containing genes in Ler ecotype^31^. However, the function of TEK in relation to pollen development has not been investigated. Our results demonstrate that *TEK* is essential for nexine formation. The members of the AHL family, including AHL16 (TEK), AHL21 (GIK), AHL25 (AGF1) and AHL15 (AGF2), were reported to maintain genome integrity. They also act as a molecular node to regulate the downstream gene expression through binding to the *cis*-acting sequence^44^,^45^. TEK is likely to regulate the expression of genes essential for the nexine formation. The identification of these target genes of TEK should facilitate the understanding of the nexine composition and function.

Recent investigations revealed that several tapetal-specific genes participated in exine formation, such as ACOS5, CYP703A2/704B1, TKPR1/2, LAP5/6 and MS2 for sporopollenin biosynthesis^16^,^17^,^18^,^19^,^20^,^21^,^22^ and ABCG26 for precursor transportation^27^,^28^,^29^. However, the molecular mechanisms how these tapetal genes regulate exine development are not clear. As regulatory genes were identifed in tapetal genetic pathway (DYT1-TDF1-AMS-MS188-MS1), the identification of other pollen wall-related genes would further reveal elaborate mechanism for tapetum to control the synthesis and transportation of exine materials. In this work, we found that AMS can directly bind to *TEK* promoter, showing that the regulatory pathway (*DYT1*-*TDF1*-*AMS*-*TEK*) in tapetum is required for nexine layer formation. On the other hand, *MS188* is the key regulator for sexine formation. Therefore, the direct binding of AMS to *MS188* promoter represents the tapetal regulatory pathway (*DYT1*-*TDF1*-*AMS*-*MS188*) for sexine layer formation. These two genetic pathways in the tapetum strongly confirm the previous studies that exine, including both sexine and nexine, is sporophytically controlled by the tapetum^8^,^9^,^10^,^11^,^12^.

**Figure 6:**
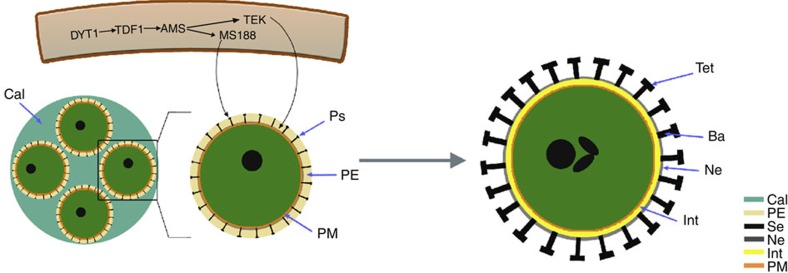
Diagrammatic representation of the pollen wall development. The AMS activated the expression of *MS188* and *TEK* in tapetum. In tetrad, four microspores were surrounded by callose. The materials produced in tapetum were transported to form the pro-sexine and nexine in the primexine. The mature pollen wall contains sexine (bacula and tectum), nexine and intine layers. Ba, bacula; Cals, callose; Int, intine; Ne, nexine; PE, primexine; PM, plasmamembrane; Ps, pro-sexine; SE, sexine; Tet, tectum.

The molecular cloning of *TEK* and identification of the genetic pathways for pollen wall formation allow us to further understand the pollen wall development (Fig. 6). As both the key regulators Male Sterile 188 (MS188) and TEK for sexine and nexine formation, respectively, are directly targeted by AMS, the formation of sexine and nexine probably initiates at the same time inside the tetrad. TEM analysis showed the absence of sexine in *ms188* mutant with normal nexine layer^11^ and the normal sexine in *tek* mutant without nexine. In addition, the expression of *MS188* is independent of *TEK*. All these suggest that these two layers are independently formed, although both of them are under regulation of AMS. The nexine layer is already formed when microspores are released from the tetrad, while sporopollenin continues to accumulate outside the nexine to form the mature sexine. The intine is formed after the nexine, which can be observed at stage 10. We propose that the nexine may play a role in protecting the released microspore until the intine is formed and act as a template for the accumulation of materials during intine formation.

## Methods

### Plant material

*Arabidopsis*, ecotype Columbia-0, was the source of both the wild-type and mutant plants. Plants were grown under long-day conditions (16-h of light/8-h of dark) in a growth room at ~22 °C. The *tek* mutant was isolated from T-DNA lines provided by Dr Zu-hua He. The herbicide Basta was used to monitor the segregation of the T-DNA inserts.

### Microscopy

Plants were photographed with a Cybershot T-20 digital camera (Sony, Japan). Flower images were taken using a dissecting microscope with an DP70 digital camera (Olympus, Japan). Alexander’s solution was made by the following reagents^46^: ethanol, 10 ml; 1% malachite green in 95% ethanol, 1 ml; distilled water, 50 ml; glycerol 25 ml; phenol, 5 g; chloral hydrate, 5 g; acid fuchsin 1% in water, 5 ml; orange G, 1% in water 0.5 ml and glacial acetic acid, 1–4 ml. Staining the anthers overnight at room temperature. Plant material for the semi-thin sections was prepared and embedded in Spurr’s resin^47^. Semi-thin sections were prepared by cutting the plant material into 1-μm thick sections, and then were stained and photographed by bright-field microscopy. For intine layer observation, the sections were stained with Toluidine blue 5 min (10 mg ml^−1^), Tinapol 15 min (10 μg ml^−1^) (Sigma, USA) and DiOC_2_ 5 min (5 μl ml^−1^) (Sigma). For scanning electron microscopic examination, fresh pollen grains were coated with 8 nm of gold and observed under JSM-840 microscopy (JEOL, Japan). For TEM observation, the same-stage buds of wild type and mutant grown at the same condition were dissected to avoid experimental deviation. *Arabidopsis* buds from the inflorescence were fixed in 0.1 M phosphate buffer (pH 7.2) with 2.5% glutaraldehyde (v/v), then washed several times before being dehydrated through a series of acetone/water mixtures. Finally, the flower buds were embedded into the fresh mixed resin and polymerized in molds (60 °C, 24 h)^37^. Ultrathin sections (70–100 nm thick) were observed using TEM microscopy (JEOL, Japan). Green fluorescent protein (GFP) fluorescence was detected under an IX70 inverted microscope system (Olympus).

### Identification and complementation of the *TEK* gene

The identification of the T-DNA insertion was performed using primers that specifically amplify the *BAR* gene of T-DNA (*Bar-F* and *Bar-R*). For TAIL–PCR, the T-DNA left border primer (AtLB1, AtLB2 and AtLB3), and genomic DNA of the mutant plants were used. Identification of the T-DNA insertion site was analysed with AtLB3 and plant-specific primers (ILP and IRP). A DNA fragment of 2,277 bp, including 725 bp upstream and 778 bp downstream sequences, was amplified by primers of CLP-F and CRP-R using KOD polymerase (Takara Biotechnology, Japan). The fragment was cloned into a pCAMBIA1300 binary vector (CAMBIA, Australia) and verified by sequencing. The plasmids were transformed into *Agrobacterium tumefaciens* GV3101 and then introduced into the heterozygous mutant plants. Transformants were selected using 20 mg l^−1^ hygromycin. The backgrounds of the transformants were verified by primers of AtLB3, IRP, CLPV-F and CRPV-R. Oligonucleotide sequences are provided in Supplementary Table 2.

### Expression analysis and *in situ* hybridization

Full-length complementary DNA of the *TEK* cloned from wild-type plants without the stop codon was cloned for the GFP fusion with primers GFP-F and GFP-R. The PCR product was cloned into the PMON530 vector with enhanced GFP and transformed into newly formed tobacco leaves with injection^48^; the GFP fluorescence of transgenic plants was observed with a Zeiss confocal laser scanning microscope. For RT–PCR, RNA was extracted from the root, stem, rosette leaves, 21-day-old seedlings and inflorescences using a Trizol kit (Invitrogen, USA). RT–PCR for 28 cycles was used to analyse the expression level of the *TEK* gene by primers (RTTEK-F and RTTEK-R). Non-radioactive RNA *in situ* hybridization was performed with using the Digoxigenin RNA Labeling Kit (Roche, USA) and the PCR DIG Probe Synthesis Kit (Roche). A 450-bp *TEK* cDNA fragment was amplified using *TEK*-specific primers (TEK-F and TEK-R). A 417-bp fragment was amplified using *AtMYB103*-specific primers (MS188-F and MS188-R). A 334-bp fragment was amplified using *AtUSP*-specific primers (USP-F and USP-R). The PCR products were cloned into the pbluescriptSK vector and confirmed by sequencing. Plasmid DNA were completely digested and used as the template for transcription with T3 or T7 RNA polymerase, respectively^49^. Oligonucleotide sequences are provided in Supplementary Table 2.

### Microarrays

Closed buds collected from wild-type and *tek* mutant plants were immediately frozen in liquid nitrogen. Three biological replicates of independently grown materials were used. Total RNA was amplified and labelled by Low RNA Input Linear Amplification kit (catalogue number 5184-3523, Agilent Technologies, USA), 5-(3-aminoallyl)-UTP (catalogue number AM8436, Ambion, USA), Cy3 NHS ester (catalogue number PA13105, GE Healthcare Biosciences, USA), Cy5 NHS ester (catalogue number PA15100, GE Healthcare Biosciences) followed the manufacturer’s instructions. Labelled cRNA were purified by RNeasy mini kit (catalogue number 74106, QIAGEN, Germany). Each 44 K *Arabidopsis* oligo microarray slide was hybridized with 825 ng Cy3-labelled cRNA and 825 ng Cy-5 labelled cRNA using Gene Expression Hybridization Kit (catalogue number 5188-5242, Agilent Technologies) in Hybridization Oven (catalogue number G2545A, Agilent Technologies), according to the manufacturer’s instructions. Slides were scanned by Agilent Microarray Scanner (catalogue number G2565BA, Agilent Technologies) and Feature Extraction software 10.7 (Agilent Technologies) with default settings. Raw data were normalized by Lowess (locally weighted scatter plot smoothing) algorithm, Gene Spring Software 11.0 (Agilent Technologies). The spots that displayed an expression ratio (*tek*/wild type) of <0.5 (for downregulated genes) and >2 (for upregulated genes), a Signal/Noise value >2.6 and a pValueLogRatio <0.05 in three independent experiments were selected.

### Chromatin immunoprecipitation

ChIP assay was performed according to ref. 50 with minor modifications. A total of 0.8–1.0 g inflorescence of wild-type plant were collected and crosslinked in the buffer containing formaldehyde. After isolating the nuclei and shearing the chromatin with ultrasonic, the majority of the DNA fragments have a size between 200–800 bp. After pre-immuneserum with sheared salmon sperm DNA/proteinA agarose mix (Millipore, USA) for 1 h, the supernatants were incubated with polyclonal antibody against AMS (GL Biochem, China) at 4 °C overnight with 1:100 dilution. Forty microlitres of magnetic beads protein G (Invitrogen) were added to precipitate the antibody–protein/DNA complexes. The DNA fragments were eluted after reverse crosslinking by boiling at 100 °C for 10 min. The remaining steps for purification of DNA were carried out according to the manufacturer’s instructions. Real-time PCR were performed on an ABI PRISM 7300 detection system (Applied Biosystems, USA) with SYBR Green I master mix (TOYOBO, Japan)^51^. All PCR experiments were performed as the following conditions: 95 °C for 5 min, 40 cycles of 95 °C for 10 s and 62 °C for 1 min. Under the same conditions, we calculated the *Δ*Ct values (Ct of each sample−Ct of the NoAb control) and took 2^−*Δ*Ct^ as the fold enrichment. The relevant primers were listed in Supplementary Table 2.

### Electrophoretic mobility shift assay

To obtain purified AMS protein for the EMSA experiments, the full-length fragment of the *AMS* gene was amplified using the primer pairs AMSpMAL-F and AMSpMAL-R, and cloned into the pMAL-p5X vector (NEB, USA) to produce the (Maltose Binding Protein) MBP-AMS construct. Expression and purification of the fusion protein were performed according to the manufacturer’s instructions. The DNA fragment containing the E-box (CANNTG) in the *TEK* and *MS188* regulatory region were generated by specific primers (ETEK-F/ETEK-R and EMS188-F/EMS188-R) that were used to generate a biotin-labelled and competitor probe, respectively. The EMSA assay was performed with a LightShift Chemiluminescent EMSA Kit (Thermo Scientific, USA). Reactions were performed in binding buffer (10 mM Tris-HCl, pH 7.5, 50 mM KCl, 1 mM dithiothreitol), at room temperature for 20 min. The subsequent processes were performed according to the manufacturer’s instructions. The image was caught by Tanon-5500 Chemiluminescent Imaging System (Tanon, China).

### Protein structure prediction and phylogenetic analysis

The *TEK* protein sequence was used to search for *TEK* homologues using BLAST. Multiple sequence alignment of full-length protein sequences was performed using ClustalX 2.0. Phylogenetic trees were constructed and tested by MEGA3.1 based on the neighbour-joining method ( http://www.megasoftware.net/).

## Author contributions

The project leader is Z.-N.Y. X.-F.U. contributed Figs 1, 2a–n,p, 3, 4a, 5a. Y.L. and X.-F.X. contributed Figs 4b,c, 5b,c. J.-N.G. contributed Fig. 2p and construction of the MBP-AMS fusion plasmid. J.Z. contributed Fig. 6. X.-F.X., J.Z. and Y.L. designed the experiments. Y.L. and J.Z. wrote the paper together. S.B. contributed to interpretation and discussion of the observations.

## Additional information

**Accession codes**: Microarray data has been deposited in GEO repository under accession number GSE56497.

**How to cite this article:** Lou, Y. *et al.* The tapetal AHL family protein TEK determines nexine formation in the pollen wall. *Nat. Commun.* 5:3855 doi: 10.1038/ncomms4855 (2014).

## Supplementary Material

Supplementary Information Supplementary Figures 1-4, Supplementary Tables 1-2 and Supplementary References

## References

[b1] Scott R. J. in:Molecular and Cellular Aspects of Plant Reproduction eds Scott R. J., Stead M. A. 49–81University Press (1994).

[b2] Zinkl G. M., Zwiebel B. I., Grier D. G. & Preuss D. Pollen-stigma adhesion in *Arabidopsis*: a species-specific interaction mediated by lipophilic molecules in the pollen exine. Development 126, 5431–5440 (1999). 10556067 10.1242/dev.126.23.5431

[b3] Ariizumi T. *et al.* Disruption of the novel plant protein NEF1 affects lipid accumulation in the plastids of the tapetum and exine formation of pollen, resulting in male sterility in Arabidopsis thaliana. Plant J. 39, 170–181 (2004). 15225283 10.1111/j.1365-313X.2004.02118.x

[b4] Blackmore S., Wortley A. H., Skvarla J. J. & Rowley J. R. Pollen wall development in flowering plants. New Phytol. 174, 483–498 (2007). 17447905 10.1111/j.1469-8137.2007.02060.x

[b5] Guilford W. J., Schneider D. M., Labovitz J. & Opella S. J. High resolution solid state C NMR spectroscopy of sporopollenins from different plant taxa. Plant Physiol. 86, 134–136 (1988). 16665854 10.1104/pp.86.1.134 PMC1054442

[b6] Scott R. J., Spielmana M. & Dickinsonb H. G. Stamen structure and function. Plant Cell 16, 46–60 (2004). 10.1105/tpc.017012 PMC2643399 15131249

[b7] Heslop-Harrison J. Origin of exine. Nature 195, 1069–1071 (1962).

[b8] Dickinson H. G. & Heslop-Harrison J. Common mode of deposition for the sporopollenin of sexine and nexine. Nature 220, 926–927 (1968). 5722147 10.1038/220926a0

[b9] Mariani C., De Beuckeleer M., Truettner J., Leemans J. & Goldberg R. B. Induction of male sterility in plants by achimeric ribonuclease gene. Nature 347, 737–741 (1990).

[b10] Piffanelli P., Ross J. H. E. & Murphy D. J. Biogenesis and function of the lipidic structures of pollen grains. Sex Plant Reprod. 11, 65–80 (1998).

[b11] Zhang Z. B. *et al.* Transcription factor AtMYB103 is required for anther development by regulating tapetum development, callose dissolution and exine formation in *Arabidopsis*. Plant J. 52, 528–538 (2007). 17727613 10.1111/j.1365-313X.2007.03254.x

[b12] Xu J. *et al.* The ABORTED MICROSPORES regulatory network is required for postmeiotic male reproductive development in *Arabidopsis thaliana*. Plant Cell 22, 91–107 (2010). 20118226 10.1105/tpc.109.071803 PMC2828693

[b13] Erdtman G. Pollen walls and angiosperm phylogeny. Bot. Notiser. 113, 41–45 (1960).

[b14] Hesse M. *et al.* Pollen terminology: An Illustrated Handbook Springer (2009).

[b15] Weber M. & Ulrich S. The endexine: a frequently overlooked pollen wall layer and simple method for detection. Grana 49, 83–90 (2010).

[b16] de Azevedo Souza C. *et al.* A novel fatty Acyl-CoA synthetase is required for pollen development and sporopollenin biosynthesis in *Arabidopsis*. Plant Cell 21, 507–525 (2009). 19218397 10.1105/tpc.108.062513 PMC2660628

[b17] Morant M. *et al.* CYP703 is an ancient cytochrome P450 in land plants catalyzing in-chain hydroxylation of lauric acid to provide building blocks for sporopollenin synthesis in pollen. Plant Cell 19, 1473–1487 (2007). 17496121 10.1105/tpc.106.045948 PMC1913723

[b18] Dobritsa A. A. *et al.* CYP704B1 is a long-chain fatty acid omega-hydroxylase essential for sporopollenin synthesis in pollen of *Arabidopsis*. Plant Physiol. 151, 574–589 (2009). 19700560 10.1104/pp.109.144469 PMC2754625

[b19] Dobritsa A. A. *et al.* LAP5 and LAP6 encode anther-specific proteins with similarity to chalcone synthase essential for pollen exine development in *Arabidopsis*. Plant Physiol. 153, 937–955 (2010). 20442277 10.1104/pp.110.157446 PMC2899912

[b20] Chen W. *et al.* *Male Sterile2* encodes a plastid-localized fatty acyl carrier protein reductase required for pollen exine development in *Arabidopsis*. Plant Physiol. 157, 842–853 (2011). 21813653 10.1104/pp.111.181693 PMC3192575

[b21] Grienenberger E. *et al.* Analysis of TETRAKETIDE α-PYRONE REDUCTASE function in *Arabidopsis thaliana* reveals a previously unknown, but conserved, biochemical pathway in sporopollenin monomer biosynthesis. Plant Cell 22, 4067–4083 (2010). 21193572 10.1105/tpc.110.080036 PMC3027178

[b22] Kim S. S. *et al.* LAP6/POLYKETIDE SYNTHASE A and LAP5/POLYKETIDE SYNTHASE B encode hydroxyalkyl-pyrone synthases required for pollen development and sporopollenin biosynthesis in *Arabidopsis thaliana*. Plant Cell 22, 4045–4066 (2010). 21193570 10.1105/tpc.110.080028 PMC3027170

[b23] Ma H. Molecular genetic analyses of microsporogenesis and microgametogenesis in flowering plants. Annu. Rev. Plant Biol. 56, 393–434 (2005). 15862102 10.1146/annurev.arplant.55.031903.141717

[b24] Zhu J., Lou Y., Xu X. & Yang Z. N. A genetic pathway for tapetum development and function in *Arabidopsis*. J. Integr. Plant Biol. 53, 892–900 (2011). 21957980 10.1111/j.1744-7909.2011.01078.x

[b25] Zhang W. *et al.* Regulation of *Arabidopsis* tapetum development and function by DYSFUNCTIONAL TAPETUM1 (DYT1) encoding a putative bHLH transcription factor. Development 133, 3085–3095 (2006). 16831835 10.1242/dev.02463

[b26] Zhu J. *et al.* Defective in Tapetal Development and Function 1 is essential for anther development and tapetal function for microspore maturation in *Arabidopsis*. Plant J. 55, 266–277 (2008). 18397379 10.1111/j.1365-313X.2008.03500.x

[b27] Quilichini T. D., Friedmann M. C., Samuels A. L. & Douglas C. J. ATP-binding cassette transporter G26 is required for male fertility and pollen exine formation in *Arabidopsis*. Plant Physiol. 154, 678–690 (2010). 20732973 10.1104/pp.110.161968 PMC2949020

[b28] Choi H. *et al.* An ABCG/WBC-type ABC transporter is essential for transport of sporopollenin precursors for exine formation in developing pollen. Plant J. 65, 181–193 (2011). 21223384 10.1111/j.1365-313X.2010.04412.x

[b29] Dou X. Y. *et al.* WBC27, an adenosine tri-phosphate-binding cassette protein, controls pollen wall formation and patterning in *Arabidopsis*. J. Integr. Plant Biol. 53, 74–88 (2011). 21205178 10.1111/j.1744-7909.2010.01010.x

[b30] Qin G. *et al.* Obtaining and analysis of flanking sequences from T-DNA transformants in *Arabidopsis*. Plant Sci. 165, 941–949 (2003).

[b31] Xu Y. *et al.* A matrix protein silences transposons and repeats through interaction with retinoblastoma-associated proteins. Curr. Biol. 23, 345–350 (2013). 23394836 10.1016/j.cub.2013.01.030

[b32] Sanders P. M. *et al.* Anther developmental defects in *Arabidopsis thaliana* male-sterile mutants. Sex Plant Reprod. 11, 297–322 (1999).

[b33] Heslop-Harrison J. Wall pattern formation in angiosperm microsporogenesis. Symp. Soc. Exp. Biol. 25, 277–300 (1971). 4940549

[b34] Sheldon J. M. & Dickinson H. G. Determination of patterning in the pollen wall of *Lilium henryi*. J. Cell Sci. 63, 191–208 (1983). 6313711 10.1242/jcs.63.1.191

[b35] Blackmore S. & Barnes S. Pollen wall morphogenesis in *Tragopogon porrifolius* (Compositae: Lactuceae) and its taxonomic significance. Rev. Palaeobot. Palynol. 52, 233–246 (1987).

[b36] Southworth D. & Jernstedt J. A. Pollen exine development precedes microtubule rearrangement in *Vigna unguiculata* (Fabaceae): a model for pollen wall patterning. Protoplasma 187, 79–87 (1995).

[b37] Chang H. S. *et al.* No primexine and plasma membrane undulation is essential for primexine deposition and plasma membrane undulation during microsporogenesis in *Arabidopsis*. Plant Physiol. 158, 264–272 (2012). 22100644 10.1104/pp.111.184853 PMC3252091

[b38] Li J., Yu M., Geng L. L. & Zhao J. The fasciclin-like arabinogalactan protein gene, FLA3, is involved in microspore development of *Arabidopsis*. Plant J. 64, 482–497 (2010). 20807209 10.1111/j.1365-313X.2010.04344.x

[b39] Huang L. *et al.* The polygalacturonase gene BcMF2 from *Brassica campestris* is associated with intine development. J. Exp. Bot. 60, 301–313 (2009). 19039102 10.1093/jxb/ern295 PMC3071776

[b40] Schnurr J. A., Storey K. K., Jung H. J., Somers D. A. & Gronwald J. W. UDP-sugar pyrophosphorylase is essential for pollen development in *Arabidopsis*. Planta 224, 520–532 (2006). 16557401 10.1007/s00425-006-0240-1

[b41] Liu Y. G., Mitsukawa N., Oosumi T. & Whittier R. F. Efficient isolation and mapping of *Arabidopsis thaliana* T-DNA insert junctions by thermal asymmetric interlaced PCR. Plant J. 8, 457–463 (1995). 7550382 10.1046/j.1365-313x.1995.08030457.x

[b42] Aravind L. & Landsman D. AT-hook motifs identified in a wide variety of DNA-binding proteins. Nucleic Acids Res. 26, 4413–4421 (1998). 9742243 10.1093/nar/26.19.4413 PMC147871

[b43] Fujimoto S. *et al.* Identification of a novel plant MAR DNA binding protein localized on chromosomal surfaces. Plant Mol. Biol. 56, 225–239 (2004). 15604740 10.1007/s11103-004-3249-5

[b44] Ng K. H., Yu H. & Ito T. AGAMOUS controls GIANT KILLER, a multifunctional chromatin modifier in reproductive organ patterning and differentiation. PLoS Biol. 7, e1000251 (2009). 19956801 10.1371/journal.pbio.1000251 PMC2774341

[b45] Matsushita A., Furumoto T., Ishida S. & Takahashi Y. AGF1, an AT-hook protein, is necessary for the negative feedback of AtGA3ox1 encoding GA 3-oxidase. Plant Physiol. 143, 1152–1162 (2007). 17277098 10.1104/pp.106.093542 PMC1820926

[b46] Alexander M. P. Differential staining of aborted and nonaborted pollen. Stain Technol. 44, 117–122 (1969). 4181665 10.3109/10520296909063335

[b47] Owen H. ,A. & Makaroff C. A. Ultrastructure of microsporogenesis and microgametogenesis in *Arabidopsis thaliana* (L.) Heynh. ecotype Wassilewskija (*Brassicaceae*). Protoplasma 185, 7–21 (1995).

[b48] Vionnet M. & Rostan O. [Idiopathic spontaneous hemoperitoneum]. Swiss Surg. 9, 184–186 (2003). 12974176 10.1024/1023-9332.9.4.184

[b49] Guan Y. F. *et al.* RUPTURED POLLEN GRAIN1, a member of the MtN3/saliva gene family, is crucial for exine pattern formation and cell integrity of microspores in *Arabidopsis*. Plant Physiol. 147, 852–863 (2008). 18434608 10.1104/pp.108.118026 PMC2409014

[b50] Bowler C. *et al.* Chromatin techniques for plant cells. Plant J. 39, 776–789 (2004). 15315638 10.1111/j.1365-313X.2004.02169.x

[b51] Yang J. *et al.* AUXIN RESPONSE FACTOR17 is essential for pollen wall pattern formation in *Arabidopsis*. Plant Physiol. 162, 720–731 (2013). 23580594 10.1104/pp.113.214940 PMC3668065

