# Supplementary material for: The tapetal AHL family protein TEK determines nexine formation in the pollen wall
Source: Nat Commun. 2014 May 8;5:3855. doi: 10.1038/ncomms4855 (PMC4024750; doi:10.1038/ncomms4855)
Supplement: Supplementary Information — Supplementary Figures 1-4, Supplementary Tables 1-2 and Supplementary References [file ncomms4855-s1.pdf]

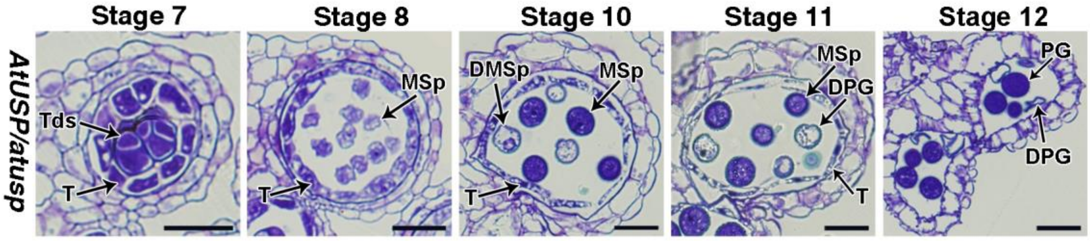

Supplementary Figure 1. Semi-thin sections of *atusp*/+ plant.

Half microspores in *atusp*/+ locule are defective. T, tapetum; Tds, tetrads; MSp, microspore; DMSp, degenerated microspores; PG, pollen grain; DPG, degenerated pollen grain. Bars=20 um.

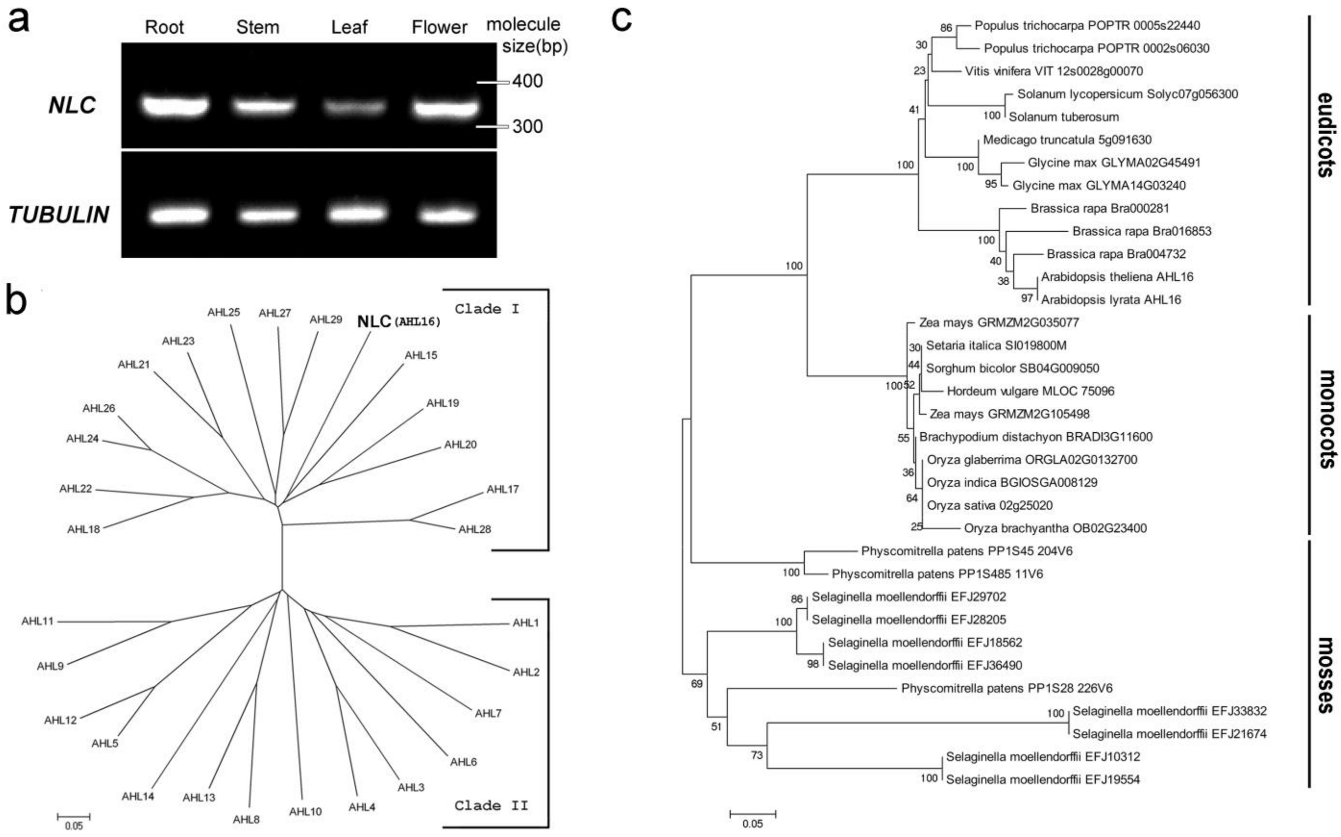

Supplementary Figure 2. Characterization of *NLC* gene.

a, *NLC* is widely expressed in root, stem, leaf and flower tissues by RT-PCR analysis.

b, Phylogenetic tree of the Arabidopsis *NLC* homologues distinguishing two clade based on different type of AT-hook motifs.

c, Phylogenetic tree of *NLC* homologues. Sequences from http://plants.ensembl.org/index.html are aligned and used to construct an unrooted maximum likelihood tree by MEGA3.1. Bar = 0.05 amino acid substitutions.

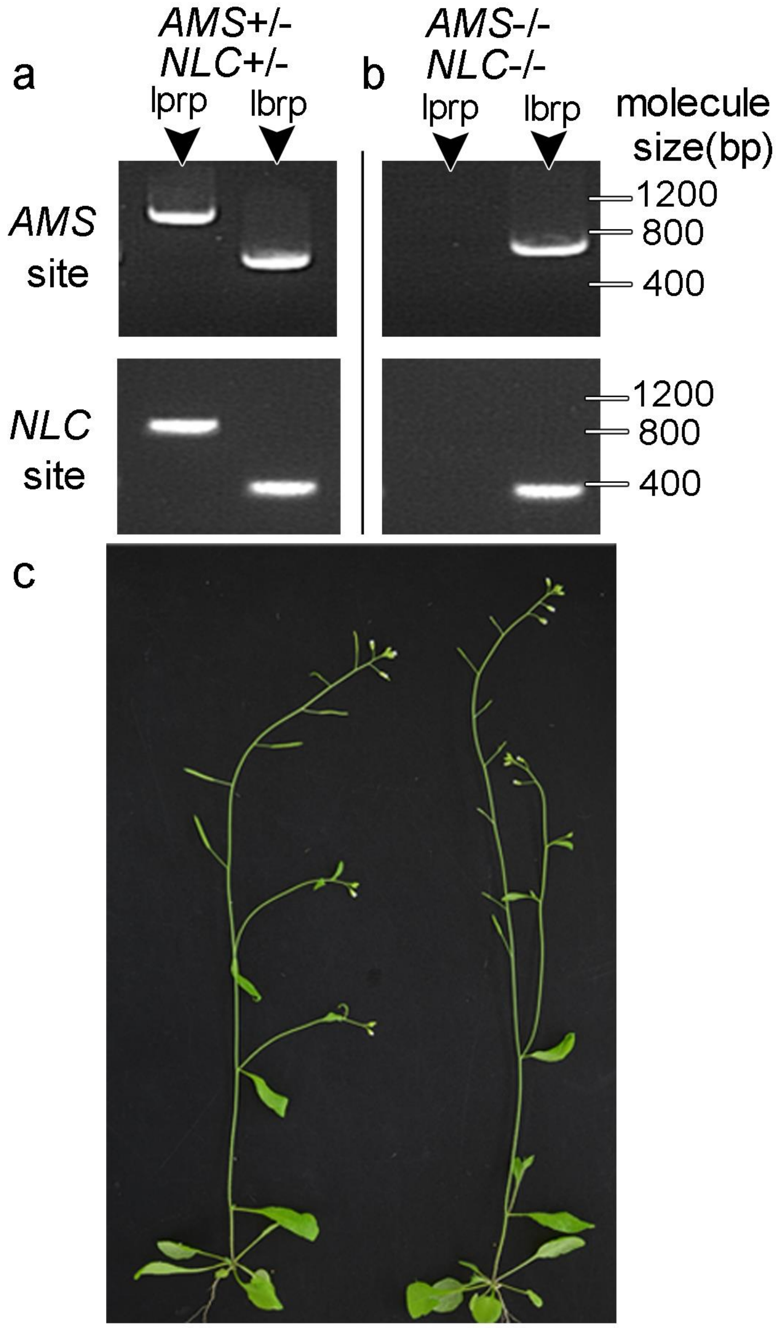

Supplementary Figure 3. Identification of *ams nlc* double mutant plant.

a, Genotyping of F1 heterozygous plant of *ams nlc* double mutant.

b, Genotyping of *ams nlc* homozygous plant.

c, *ams nlc* is the sterile plant with normal vegetable growth.

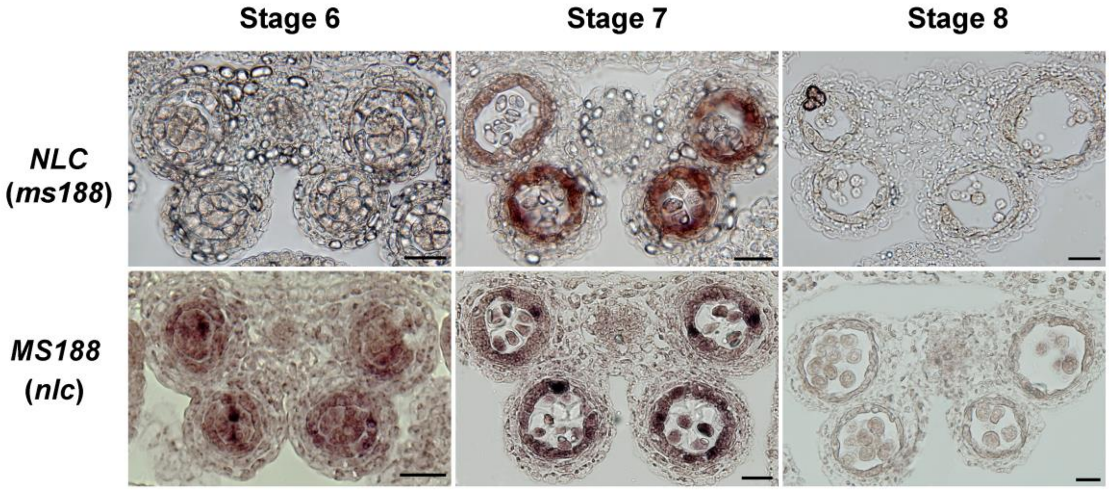

Supplementary Figure 4. Expression patterns of *NLC* and *MS188* in *ms188* and *nlc* mutant background, respectively.

Expression pattern of *NLC* is not affected in *ms188*, meanwhile expression pattern of *MS188* is not affected in *nlc*. Bars=20um.

Supplementary Table 1. Genes expression in *nlc* buds compared with the wild type and pollen wall integrality in mutants

Function path	GeneName	AtID	Flod Chang of Log2	Pollen Wall Integrality			References
				sexine	nexine	intine	
sporopollenin synthesis	*AMS*	At2G16910	0.46 ± 0.28	－	－	－	1
	MS188	At5G56110	0.51 ± 0.07	－	＋	＋	2
	ACOS5	At1G62940	0.94 ± 0.17	－	※	＋	3
	CYP703A2	At1G01280	0.99 ± 0.17	－	※	＋	4
	CYP704B1	At1G69500	0.70 ± 0.06	－	＋	＋	5
	TKRP1	At4G35420	0.93 ± 0.16	－	＋	?	6
	MS1	At5G22260	-0.73 ± 0.22	※	＋	?	7,8
	MS2	At3G11980	0.17 ± 0.23	－	＋	?	9
	LAP3	At3G59530	0.72 ± 0.21	※	＋	＋	10
	LAP5/PKSB	At4G34850	1.16 ± 0.20	※	＋	＋	11,12
	LAP6/PKSA	At1G02050	0.73 ± 0.07	※	＋	＋	11,12
	FLP1	At5G57800	0.19 ± 0.34	※	＋	＋	13
transport protein	*ABCG26*	At3G13220	1.13 ± 0.40	－	＋	＋	14,15
intine synthesis	*AtUSP*	At5G52560	0.28 ± 0.12	＋	＋	－	16
	FLA3	At2G24450	-6.06 ± 0.29	＋	＋	－	17

Pollen wall integrity of each mutant is marked +, normal; －, absent; ※, abnormal; ?, unknown.

Gene expressions are used log_2-transformed expression ratios (±SD) from three independent hybridization slides.

The references are orderly listed in the Supplementary figure legend.

Supplementary Table 2. List of primers used in the study and their sequences

Prime	Sequence	Note
Bar-F	5'-GCACCATCGTCAACCACTAC-3'	Amplifiying the BAR gene for indentify the T-DNA insertion
Bar-R	5'-TGCCAGAAACCCACGTCAT-3'	
AtLB1	5'-ATACGACGGATCGTAATTTGTC-3'	For Tail-PCR
AtLB2	5'-TAATAACGCTGCGGACATCTAC-3'	
AtLB3	5'-TTGACCATCATACTCATTGCTG-3'	
ILP	5'– ATAACAATGGCTGGAGGTACAG-3'	Identifiying the T-DNA insertion site and mutant phenotype
IRP	5'-GAAACGTGGAGATTAG AGCAG TAG -3'	
CLP-F	5'-AACAATCTCGAAATTTTAGGC-3'	For complementation
CRP-R	5'-CGTGAGGTGCAAGGAGAA-3'	
CLPV-F	5'- CTGAGAGCATTACCCAAAGC -3'	Verifiying the background of the transformants
CRPV-R	5'-TTATATCATTGCCTGGAG ACG-3'	
GFP-F	5'-ATGGCTGGAGGTACAGCTCT-3'	For p35S:*NLC*-GFP fusion
GFP-R	5'-AGGTTTCGACATGACA CGC-3'	
RTNLC-F	5'-AAGAACAAACCCAAACCACC-3'	For Real Time-PCR
RTNLC-R	5'-AACAACAGGACCAGATGCG-3'	
Tublin-F	5'-GATTTCAAAGATTAGGGAAGAGTA-3'	
Tublin-R	5'-GTTCTGAAGCAAATGTCATAGAG-3'	
AMSpMAL-F	5'-GGATCCATGGAGAGTAATATGCAAAACTTG-3'	For recombinant MBP-AMS protein
AMSpMAL-R	5'-CTGCAGTTATTGGTTGTGGTAATGGTTGA-3'	
NLC-F	5'-TCGGATTTTGCAAGAAGGA-3'	For non-radioactive RNA in situ hybridization
NLC-R	5'-CCAAGAGTAGATATCAGA AGCC-3'	
MS188-F	5'-GATGTGGGAAGAGTTGTAGGC-3'	
MS188-R	5'-GAAAGTTGTTTGGGTTAGG GT-3'	
USP-F	5'-TCTGGTTGCTGGTGGTC-3'	For qChIP-PCR
USP-R	5'-TACTGTATTTGTTGTGAGGGTCT-3'	
P1-F	5'-GGCACAGGTCGAGGACGA-3'	
P1-R	5'-CCACTGCTCTGTATTTTATCGC-3'	
P2-F	5'-GATTTTAGTTTTGGTCCCAAAAAG-3'	
P2-R	5'-CAAATTTATTTTGCAAAAAAAGAA-3'	
P3-F	5'-CTCCTACTCCTCACAATCATTCTTT-3'	
P3-R	5'-TGTTATGAATGTTGTTATATGTTCAACT-3'	
S1-F	5'-AAGTTGTGTTTTTTCCCAAGTCA-3'	
S1-R	5'-CCATCCCCCACAACTTGTG-3'	
S2-F	5'-CAGAGAAACTGAAACTAATTTTCCA-3'	
S2-R	5'-CTTGAATATCGATCAAAATGTAAATATA-3'	
S3-F	5'-GGAGTTGACCAGGCGTTGA-3'	
S3-R	5'-AACAAAAATGAAAACATAGTAAAAATT-3'	
ENLC-F	5' AGCATTATTATGAATCTCTCTGTTA 3'	For EMSA

ENLC-R	5'	TTGTTATATGTTCAACTGAAAGATT	3'
EMS188-F	5'	CAGAGAAACTGAAACTAATTTTCCA	3'
EMS188-R	5'	GAATTTGAAAATTAGATGAGAGACA	3'

Supplementary References:

1. Xu, J. *et al*. The ABORTED MICROSPORES regulatory network is required for postmeiotic male reproductive development in Arabidopsis thaliana. *Plant Cell* **22,** 91-107 (2010).
2. Zhang, Z.B. *et al.* Transcription factor AtMYB103 is required for anther development by regulating tapetum development, callose dissolution and exine formation in Arabidopsis. *Plant J* **52,** 528-538 (2007).
3. de Azevedo Souza C. *et al.* A novel fatty Acyl-CoA synthetase is required for pollen development and sporopollenin biosynthesis in Arabidopsis. *Plant Cell* **21,** 507–525 (2009).
4. Morant M. *et al.* CYP703 is an ancient cytochrome P450 in land plants catalyzing in-chain hydroxylation of lauric acid to provide building blocks for sporopollenin synthesis in pollen. *Plant Cell* **19,** 1473–1487 (2007).
5. Dobritsa AA. *et al.* CYP704B1 is a long-chain fatty acid omega-hydroxylase essential for sporopollenin synthesis in pollen of Arabidopsis. *Plant Physiol* **151,** 574–589 (2009).
6. Grienenberger E. *et al.* Analysis of TETRAKETIDE α-PYRONE REDUCTASE function in Arabidopsis thaliana reveals a previously unknown, but conserved, biochemical pathway in sporopollenin monomer biosynthesis. *Plant Cell* **22,** 4067–4083 (2010).
7. Ito T. *et al.* Arabidopsis MALE STERILITY1 encodes a PHD-type transcription factor and regulates pollen and tapetum development. *Plant Cell* **19,** 3549–3562 (2007).
8. Yang C, Vizcay–Barrena G, Conner K & Wilson Z.A. MALE STERILITY1 is required for tapetal development and pollen wall biosynthesis. *Plant Cell* **19,** 3530–3548 (2007).
9. Aarts M.G. *et al.* The Arabidopsis MALE STERILITY 2 protein shares similarity with reductases in elongation/condensation complexes. *Plant J* **12,** 615-23(1997).

10. Dobritsa A.A. *et al.* LAP3, a novel plant protein required for pollen development, is essential for proper exine formation. *Sex Plant Reprod* **22,** 167-77 (2009).
11. Dobritsa, A.A. *et al.* LAP5 and LAP6 encode anther-specific proteins with similarity to chalcone synthase essential for pollen exine development in Arabidopsis. Plant Physiol. **153,** 937-955 (2010).
12. Kim S.S. *et al.* LAP6/POLYKETIDE SYNTHASE A and LAP5/POLYKETIDE SYNTHASE B Encode Hydroxyalkyl-Pyrone Synthases Required for Pollen Development and Sporopollenin Biosynthesis in Arabidopsis thaliana. *Plant Cell* **22,** 4045–4066 (2010).
13. Ariizumi T. *et al.* A novel male–sterile mutant of Arabidopsis thaliana, faceless pollen-1, produces pollen with a smooth surface and an acetolysis–sensitive exine. *Plant Mol Biol* **53,** 107–116 (2003).
14. Quilichini, T.D., Friedmann, M.C., Samuels, A.L. & Douglas, C.J. ATP-binding cassette transporter G26 is required for male fertility and pollen exine formation in Arabidopsis. *Plant Physiol.* **154,** 678-690 (2010).
15. Dou, X.Y. et al. WBC27, an adenosine tri-phosphate-binding cassette protein, controls pollen wall formation and patterning in Arabidopsis. *J Integr Plant Biol* **53,** 74-88 (2011).
16. Schnurr J.A., Storey K.K., Jung H.J., Somers D.A. & Gronwald J.W. UDP-sugar pyrophosphorylase is essential for pollen development in Arabidopsis. *Planta* 224, 520–532 (2006).
17. Li J., Yu M., Geng L.L. & Zhao J. The fasciclin-like arabinogalactan protein gene, FLA3, is involved in microspore development of Arabidopsis. *Plant J* **64,** 482–497 (2010).
